# Facing the challenges in ophthalmology clerkship teaching: Is flipped classroom the answer?

**DOI:** 10.1371/journal.pone.0174829

**Published:** 2017-04-06

**Authors:** Ying Lin, Yi Zhu, Chuan Chen, Wei Wang, Tingting Chen, Tao Li, Yonghao Li, Bingqian Liu, Yu Lian, Lin Lu, Yuxian Zou, Yizhi Liu

**Affiliations:** 1 State Key Laboratory of Ophthalmology, Zhongshan Ophthalmic Center, Sun Yat-sen University, Guangzhou, Guangdong, China; 2 Department of Molecular and Cellular Pharmacology, University of Miami Miller School of Medicine, Miami, Florida, United States of America; Medizinische Universitat Graz, AUSTRIA

## Abstract

Recent reform of medical education highlights the growing concerns about the capability of the current educational model to equip medical school students with essential skills for future career development. In the field of ophthalmology, although many attempts have been made to address the problem of the decreasing teaching time and the increasing load of course content, a growing body of literature indicates the need to reform the current ophthalmology teaching strategies. Flipped classroom is a new pedagogical model in which students develop a basic understanding of the course materials before class, and use in-class time for learner-centered activities, such as group discussion and presentation. However, few studies have evaluated the effectiveness of the flipped classroom in ophthalmology education. This study, for the first time, assesses the use of flipped classroom in ophthalmology, specifically glaucoma and ocular trauma clerkship teaching. A total number of 44 international medical school students from diverse background were enrolled in this study, and randomly divided into two groups. One group took the flipped glaucoma classroom and lecture-based ocular trauma classroom, while the other group took the flipped ocular trauma classroom and lecture-based glaucoma classroom. In the traditional lecture-based classroom, students attended the didactic lecture and did the homework after class. In the flipped classroom, students were asked to watch the prerecorded lectures before the class, and use the class time for homework discussion. Both the teachers and students were asked to complete feedback questionnaires after the classroom. We found that the two groups did not show differences in the final exam scores. However, the flipped classroom helped students to develop skills in problem solving, creative thinking and team working. Also, compared to the lecture-based classroom, both teachers and students were more satisfied with the flipped classroom. Interestingly, students had a more positive attitude towards the flipped ocular trauma classroom than the flipped glaucoma classroom regarding the teaching process, the course materials, and the value of the classroom. Therefore, the flipped classroom model in ophthalmology teaching showed promise as an effective approach to promote active learning.

## Introduction

As our understanding of the pathophysiology of diseases keeps on expanding, and new treatment technology continuously emerges, the limitations of the traditional lecture-based teaching becomes obvious. First, the increasing load of course content and a decreasing teaching time due to the competing service demands pose a big challenge to the instructors[1]. Second, lecture-based teaching lacks efficiency, as previous studies show that the average attention span of the medical students during a lecture is only 10 to 20 minutes at the beginning[2]. Third, passive learning experience during the hour-long lecture inevitably bores students and deprives them from acquiring essential survival skills, such as critical thinking, problem solving and communication[3]. These growing concerns on the quality of medical education call for a reform of the traditional didactic teaching approach to better prepare students for their future career development.

Previous studies have shown that content comprehension and knowledge retention can be greatly enhanced if students are actively participated into the learning process [4, 5]. Medical school students usually have the ability to learn the information on the textbook on their own, but they need guidance from instructors for solving problems in real clinical practice [6, 7]. This leads to the rationale for using the “flipped classroom” as a potentially effective method for medical education. The flipped classroom is an educational model in which the lecture and homework elements of a course are reversed. In the flipped classroom model, students listen to podcasts or view video-recorded lectures on their own before attending the class, and use the in-class time for student-centered learning activities such as case scenario analysis [8]. In the flipped classroom approach, educators do not drive the teaching process, but devote their time to guiding collaborative learning and application of knowledge. Also, students are required to play an active role during the class rather than passively absorbing lecture materials.

The modality of flipped classroom has long been applied in non-science education, and recent development of video recording and internet capability expand this approach into teaching medical courses[9]. Johnathan, et al.[10] showed that the flipped classroom model is helpful in disseminating key concepts in cardiovascular, respiratory, and renal physiology. Although previous investigations suggest that flipped classroom improves students’ performance by promoting active learning, the overall effectiveness of this approach in medical education is still controversial. For example, Stephney, et al.[11]showed that the design of flipped classroom does not add any value to the lecture-based approach, and no differences in grades or level of satisfaction were found in a flipped neuroanatomy classroom. One possible explanation is that this model of knowledge delivery is not suitable for subjects that carry heavy and abstract content, as students need to spend too much time for preparation before the class. Therefore, the flipped classroom approach requires further research to evaluate its value before being applied in specific courses.

Ophthalmic clinical skill training is essential to all medical practitioners, as the visual system interacts with other bodily systems, and ophthalmic abnormalities can be key clues for systemic disease diagnosis. Concurrent with the increasing need for ophthalmic care providers, ophthalmology education, however, has been marginalized worldwide as a consequence of decreasing curriculum time[12]. This requires ophthalmic educators to disseminate complex information to students in a more effective way. A number of potential practices that might enhance ophthalmology teaching have been studied, such as integrated problem based learning[13]and team-based learning[14]. We have previously reported that the application of team-based learning to the ophthalmology clerkship curriculum significantly improved students’ performance[15], highlighting the importance of student engagement and interaction in learning. However, the effectiveness of the flipped classroom versus traditional lecture-based classroom in ophthalmology teaching has not been investigated worldwide.

In this study, we for the first time evaluated the effectiveness and acceptability of the flipped classroom curriculum versus lecture-based curriculum of glaucoma and ocular trauma delivering to the international medical school students at Zhongshan Ophthalmic Center (ZOC) of Sun Yat-sen University in the spring of 2016.ZOC is the first western-style hospital in China[16], and has rich human resources for ophthalmic education and training. The goal of this study is to take the first step into providing quantitative data about the effectiveness of the flipped classroom model in conveying ophthalmic knowledge to medical students. This article may serve as a guide for ophthalmic instructors to implement and develop better practices for future ophthalmology teaching.

## Methods

### Study population

Participants(n = 44) in this study were international students from diverse background enrolled in the Bachelor of Medicine and Bachelor of Surgery (MBBS) program. They had already attended all the ophthalmology lectures in Sun Yat-sen University before they went to ZOC for clinical clerkship in different ophthalmic subspecialties. Students were randomly divided into two groups. One group (n = 22) participated in the lecture-based ocular trauma classroom and flipped glaucoma classroom. The other group (n = 22) participated in the lecture-based glaucoma classroom and flipped ocular trauma classroom. All the subjects were not aware of the differences in the course format before the enrollment. All the procedures in this study were videotaped, with the approval of the institutional review board of Zhongshan Ophthalmic Centre of Sun Yat-sen University (IRB-ZOC-SYSU). Written informed consents have been obtained from all students.

### Procedure

The chronology of the two classrooms is summarized in Table 1. Both classrooms are taught in English. Since all the students had already attended ophthalmology lectures before they came to ZOC, we performed a pre-class quiz to assess the students’ baseline understandings of glaucoma and ocular trauma knowledge. The quiz contained 20 glaucoma questions and 20 ocular trauma multiple choice questions. Each question had the same weight. We calculated the total glaucoma scores and the total ocular trauma scores for each student. After the quiz, all the students in both groups were provided with essentially identical learning objectives and lecture handouts.

**Table 1:** The chronology of the lecture-based classroom and flipped classroom.

	Lecture-based classroom	Flipped classroom
Before class	Pre-test	
	• Provided learning objectives and lecture handouts	• Provided learning objectives and lecture handouts • Read the chapter of glaucoma/ ocular trauma • Watch recorded video lecture • Preparation of case report (6 slides for each group)
During class	• Listen to lecture and take notes (60 mins) • Q & A session (15 mins)	• Brief introduction of the case by the instructor (15 mins) • Case presentation by each group & discussion (45 mins) • Summary of the class, Q & A session (15 mins)
After class	• Review the chapter of glaucoma/ocular trauma • Homework (same case and questions discussed in the flipped classroom) • Receive feedback from the instructors	• Fill out feedback questionnaire
	Final exam	

In the lecture-based classroom, the students attended a 60-min traditional didactic lecture followed by a 15-minute question and answer session led by the instructor. To assess the understanding of the lecture contents by the students, a homework assignment was posted onto the blackboard after the lecture. In order to reinforce the knowledge, the students were encouraged to briefly review the chapter of glaucoma/ocular trauma before working on their homework. The homework contained the same case and questions discussed in the flipped classroom, and the first question was to briefly summarize the major points of the case. Students were asked to hand in the homework within one week after the class, and then they received feedback from the instructor. The instructor provided the answers to each question and helped the students to work through any particularly difficult questions if the students requested help.

In the flipped classroom, students were divided into six small groups (three to four students per group). A leader was assigned for each group. All students were asked to watch the recorded video lecture online. Additionally, the instructor prepared and sent out a clinical case and related questions to be discussed in the classroom. Each small group received the same case, and was asked to prepare a six-slide PowerPoint case report for in-class presentation. For example, in the glaucoma flipped classroom, students were provided a case describing a patient presenting acute severe eye pain, accompanied by ipsilateral headache. Ophthalmic examination revealed corneal edema and mydriasis. Open-ended questions included: (1) What other ophthalmic examinations are required for diagnosis of primary angle closure glaucoma (PACG)? (2) How to alleviate pain and reduce intraocular pressure (IOP)? (3) How to make differential diagnosis between PACG, common cold, and acute gastroenteritis? (4) What clinical signs may follow after IOP is decreased? (5) What is the normal structure of the anterior chamber angle? What are the Shaffer's and Scheie's grading systems? (6) How to select the operation method according to the grading of anterior chamber angle/depth? (7) How to conduct post-operative follow-ups and evaluate the effectiveness of the surgery? In the flipped classroom, the instructor briefly recapitulated the case, after which each group presented the case and their answers to the questions. During the presentation, the other groups were encouraged to interrupt and challenge their answers. Also, the presenting group could propose new questions for further discussion among groups. All the students in the flipped classroom worked in groups to tackle real clinical cases by applying what they had learned from the textbooks and lectures. Finally, the instructor summarized the whole class, and reviewed all questions from the discussion.

To investigate the students’ and teachers’ attitudes towards the flipped classroom, all the students (n = 44) and teachers (n = 10) were asked to fill out feedback questionnaires immediately after the class. The questionnaire was adapted from Paul Ramsden’s Course Experience Questionnaire (CEQ)[17]and Biggs’ Study Process questionnaire[18]. The questionnaire contained questions regarding the learning experience, perceived value of the flipped classroom, course materials, teaching process, and the evaluation system. The questionnaire used a standard 5-point Likert scale, and the levels of agreement ranged from “strongly agree” (scoring a “1”) to “strongly disagree” (scoring a “5”). Teachers were also asked to select topics they thought were more suitable for applying the flipped classroom model, and to suggest the appropriate frequency of flipped classroom in ophthalmology clerkship teaching.

To assess the student learning and performance, a final exam was administered to all students. This exam also consisted of 20 glaucoma and 20 ocular trauma multiple choice questions. Each question had the same weight. Similar to the pre-class quiz, we calculated the total glaucoma scores and total ocular trauma scores for each student.

### Statistical analysis

The population demographic data were analyzed using Chi-square test or independent samples t test. The scores of the pre-class quiz and final exam were compared using independent samples t test. The questionnaire data were analyzed using Mann-Whitney-Wilcoxon test. All statistical analyses were performed in the SPSS statistical package (ver.22.0). P<0.05 was considered statistically significant. All data were reported as means ± S.D.

## Results

The demographic distribution of the participants was presented in Table 2. A total of 44international students were enrolled. Twenty-two students participated in the flipped glaucoma classroom and lecture-based ocular trauma classroom. Twenty-two students participated in the flipped ocular trauma classroom and lecture-based glaucoma classroom. Students were from diverse countries including India, Sri Lanka, Nepal, Singapore, Thailand, Malaysia, Ghana, Mauritius, United Kingdom, United States and Canada. There were no statistical differences between gender (x^2^ = 0.093, P = 0.761), age (t = 1.568, P = 0.124), and place of residence (x^2^ = 0.093, P = 0.761). Most of them had been in China for 5 years, and were in the 5^th^ year of medical school. None of the students withdrew from the classes, and all students completed the pre-class quiz and the final exam.

**Table 2:** Demographic information of students participated in glaucoma flipped classroom and ocular trauma flipped classroom.

	Flipped glaucoma classroom	Flipped ocular trauma classroom	P Value
Number of students	22	22	
Gender			
Male	9 (41%)	10 (45%)	0.761^a^
Female	13 (59%)	12 (55%)	
Place of residence			
Rural	10 (45%)	9 (41%)	0.761^a^
Urban	12 (55%)	13 (59%)	
Age (years)	23.5±1.1	24.2±2.2	0.124^b^
Years in China	5.0±0.2	5.0±0.7	0.574^b^
Years of leaning Chinese	1.5±1.0	1.5±0.5	1.000^b^
Years of medical school	5.2±0.5	5.0±0.5	0.143^b^

a. Pearson Chi-Square test

b. Independent samples t test

**Fig 1:**
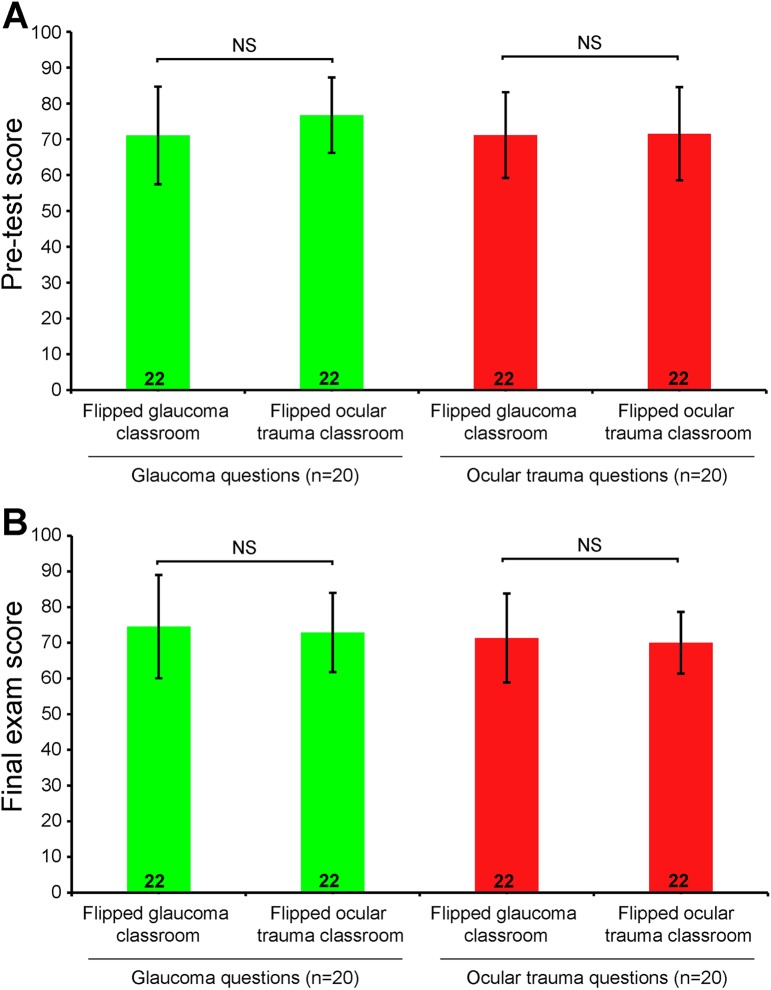
Comparison of students’ exam scores before and after the classroom. Students were asked to answer 20 glaucoma and 20 ocular trauma multiple choice questions before (A) and after (B) taking the classroom. Each question had the same weight, and the total score was converted into a 0–100 scale. Independent samples t test was used to compare the differences between the two groups. All data were presented as mean± S.D. n = 22.

In the pre-class quiz, there were no statistical differences between the two groups in either glaucoma scores (t = 0.026, P = 0.979) or ocular trauma scores (t = 1.452, P = 0.154), suggesting that the baseline understandings to glaucoma and ocular trauma in the two groups were comparable (Fig 1A). The final exam score showed that there were no statistical differences between the two groups in either glaucoma scores (t = 0.782, P = 0.439) or ocular trauma scores (t = 0.979, P = 0.334) (Fig 1B). This result indicated that the flipped classroom approach did not increase the scores of students in the final exam.

All students and teachers completed the questionnaires at the end of the session, with a response rate of 100%. Table 3 summarizes the responses from the students regarding the learning experience, the perceived value of the flipped classroom, the course materials, the teaching process and the evaluation system. Overall, students were satisfied with the flipped classroom model, and most students agreed that the climate of this class is conducive to learning. Interestingly, there were some differences between the responses of the two groups regarding the course materials, teaching process, and perceived value of the flipped classroom. Compared to the glaucoma flipped classroom, students felt that the ocular trauma flipped classroom was more helpful in terms of developing teamwork (Z = 2.19, P = 0.034) and problem-solving skills (Z = 2.06, P = 0.043). Also, compared to the ocular trauma flipped classroom, more students in the glaucoma flipped classroom tended to agree that the work load was too heavy (Z = 2.32, P = 0.025), the class was overly theoretical and abstract (Z = 2.52, P = 0.013), and they did not have a clear idea of the expectation of the course (Z = 2.45, P = 0.011).

**Table 3:** Comparison of students’ perspectives between flipped glaucoma classroom and flipped ocular trauma classroom.

	Flipped glaucoma classroom	Flipped ocular trauma classroom	P value^a^	Effect size (r)^b^
**1. Questions regarding the learning experience**				
The course met my expectations.	1.63 ± 0.83	1.57 ± 0.59	0.944	0.01
It is an enjoyable way of learning.	1.42 ± 0.60	1.39 ± 0.50	0.988	0.00
Overall, I am satisfied with the quality of this course.	1.42 ± 0.60	1.60 ± 0.66	0.324	0.15
The climate of this class is conducive to learning.	1.63 ± 0.83	1.65 ± 0.65	0.674	0.06
**2. Questions regarding the value of the flipped classroom**				
The lecture greatly enhances my learning about this topic.	1.53 ± 0.61	1.61 ± 0.58	0.608	0.08
The course developed my problem-solving skills.	1.63 ± 0.68	1.65 ± 0.49	0.719	0.06
The course sharpened my analytic skills.	1.84 ± 0.60	1.70 ± 0.56	0.434	0.12
The course helped me to develop my ability to work as a team member.	2.11 ± 0.88	1.57 ± 0.51	0.034*	0.33
As a result of my course, I feel confident about tackling unfamiliar problems.	2.16 ± 0.96	1.61 ± 0.72	0.043*	0.31
The course improved my skills in written communication.	2.32 ± 0.95	2.09 ± 0.85	0.544	0.09
My course helped me to develop the ability to plan my own work.	2.11 ± 0.66	1.78 ± 0.80	0.099	0.25
**3. Questions regarding the course materials**				
You usually have a clear idea of where you’re going and what’s expected of you in this course.	2.15 ± 0.83	1.52 ± 0.67	0.011*	0.39
It is always easy here to know the standard of work expected.	1.95 ± 0.97	1.65 ± 0.57	0.434	0.12
I was generally given enough time to understand the things we have to learn.	2.42 ± 0.77	2.43 ± 1.34	0.671	0.07
The work was too heavy.	2.58 ± 1.07	1.82 ± 1.00	0.025*	0.35
The course is overly theoretical and abstract.	2.63 ± 1.34	3.70 ± 1.22	0.013*	0.38
There was a lot of pressure on me to do well in this course.	2.42 ± 1.17	2.52 ± 1.23	0.784	0.04
The sheer volume of work to be got through in this course means you can’t comprehend it all thoroughly.	2.89 ± 0.81	2.91 ± 0.79	0.989	0.00
**4. Questions regarding the teaching process**				
The staff on this course make it clear right from the start what they expect of students.	2.21 ± 0.79	1.65 ± 0.51	0.015*	0.37
The teachers on this course motivated me to do my best work.	1.63 ± 0.68	1.47 ± 0.51	0.539	0.09
Teachers put a lot of time into commenting on student’s work.	1.68 ± 0.82	1.52 ± 0.73	0.523	0.10
Teaching staff on this course work hard to make their subjects interesting.	1.21 ± 0.42	1.47 ± 0.67	0.174	0.21
Teachers make a real effort to understand difficulties students may be having with their work.	1.68 ± 1.00	1.95 ± 0.65	0.090	0.26
Teachers normally give helpful feedback on how you’re doing.	1.95 ± 0.85	1.47 ± 0.59	0.057	0.29
Our lecturers are extremely good at explaining things to us.	1.53 ± 0.90	1.48 ± 0.59	0.692	0.06
**5. Questions regarding the evaluation system**				
Teachers seem more interested in testing what you’ve memorized than what you’ve understood.	3.11 ± 1.37	2.87 ± 1.45	0.594	0.08
Too many staff on this course ask us questions just about facts.	2.74 ± 1.15	2.77 ± 1.23	0.849	0.03
To do well in this course all you really need is a good memory.	2.68 ± 1.29	2.04 ± 0.97	0.104	0.25

a. Two groups are compared by Mann-Whitney-Wilcoxon test.

*p<0.05 is considered significant.

b. Effect size is calculated by test statistic divided by the root of sample size (small effect: 0.1 <r ≤ 0.3, medium effect: 0.3 <r ≤ 0.5, large effect: r > 0.5).

**Fig 2:**
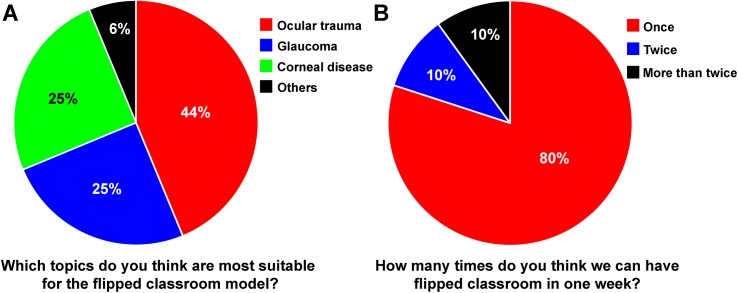
Teachers’ opinions about the topic and frequency of the flipped classroom model in ophthalmology teaching. Teachers (n = 10) were asked to pick up 1 to 3 topics that they think are most suitable to apply the flipped teaching model (A). Teachers are also asked how many times they think flipped classroom could be hold during the clerkship teaching (B).

Tables 4 and 5 summarize the feedbacks from the teachers in the glaucoma and ocular trauma clerkship teaching, respectively. All the teachers participated in the lecture-based and flipped classrooms. In ocular trauma clerkship teaching, compared to the lecture-based classroom, more teacher thought that the flipped classroom met their expectations (Z = 2.32, P = 0.032) and they were more satisfied with the quality of the flipped classroom (Z = 2.43, P = 0.032). Also, teachers agreed that the flipped classroom model was a more enjoyable way of teaching (Z = 2.45, P = 0.032), and was more conducive to learning for the students (Z = 2.63, P = 0.008). However, in glaucoma clerkship teaching, there were no significant differences in teachers’ feedback between these two teaching models. Moreover, a majority of teachers thought that ocular trauma was a more suitable topic for the flipped classroom model (Fig 2A), and the flipped classroom could be hold once during the clerkship teaching (Fig 2B).

**Table 4:** Comparison of teachers’ perspectives between the lecture-based and the flipped glaucoma classroom.

	Glaucoma classroom		P value^a^	Effect size (r)^b^
	Lecture-based	Flipped		
The lecture greatly enhances students’ understanding about this topic.	1.60 ± 0.50	1.60 ± 0.95	0.841	0.10
The course met my expectations.	2.20 ± 0.58	1.40 ± 0.58	0.151	0.70
It is an enjoyable way of teaching.	1.80 ± 0.81	1.60 ± 0.95	0.690	0.20
Overall, I am satisfied with the quality of this course.	2.00 ± 0.50	1.40 ± 1.00	0.222	0.61
The climate of this class is conducive to learning for students.	2.00 ± 0.96	1.20 ± 0.50	0.222	0.64

a. Two groups are compared by Mann-Whitney-Wilcoxon test.

b. Effect size is calculated by test statistic divided by the root of sample size.

**Table 5:** Comparison of teachers’ perspectives between the lecture-based and the flipped ocular trauma classroom.

	Ocular trauma classroom		P value^a^	Effect size (r)^b^
	Lecture-based	Flipped		
The lecture greatly enhances students’ understanding about this topic.	2.20 ± 0.45	1.60 ± 0.89	0.222	0.61
The course met my expectations.	2.60 ± 0.55	1.40 ± 0.55	0.032*	1.04
It is an enjoyable way of teaching.	2.60 ± 0.89	1.00 ± 0.00	0.032*	1.10
Overall, I am satisfied with the quality of this course.	3.20 ± 0.45	1.40 ± 0.89	0.032*	1.08
The climate of this class is conducive to learning for students.	3.00 ± 0.71	1.20 ± 0.45	0.008*	1.18

a. Two groups are compared by Mann-Whitney-Wilcoxon test.

*p<0.05 is considered significant.

b. Effect size is calculated by test statistic divided by the root of sample size.

## Discussion

This study for the first time assessed the effectiveness of flipped classroom model in the ophthalmology clerkship teaching. Based on the questionnaire feedback, we found that the flipped classroom model is highly welcomed by the participating students and faculties. Possible explanations may include: (1) Flipped classroom model is stimulating. Students’ interest in learning is stimulated by the real clinical case and open questions assigned before the flipped classroom. In contrast to reading and memorizing lecture materials in the traditional class model, students in the flipped classroom feel strongly motivated to understand and master related knowledge in order to solve real clinical problems. This increased interest on both emotional and cognitive levels is beneficial for learning[19]. (2) Flipped classroom model is student-centered. In contrast to the traditional lecture-based classroom model where teachers are centered to deliver course materials and answer questions, in the flipped classroom model, students play multiple roles including organizers of their team, presenters to visually and orally express opinions, and communicators in questioning, answering and discussing among groups. In this student-driven, teacher-facilitated approach, students are encouraged to become both instructors and learners of each other, where students feel being valued more than where they do in the traditional lecture-based classroom. Also, in this student-centered learning environment, students could break the boundaries usually set in the traditional lecture-based classroom known as “learning goals” and “syllabus”. As a result, the increase in the freedom of personalized learning goals and learning means encourages students to learn more and therefore satisfies students’ learning motivation[20]. (3) Flipped classroom model is inclusive. One challenge of the traditional lecture-based teaching is the easy loss of audience concentration. On the contrary, in the flipped classroom, every student is encouraged to contribute in the team, to effectively present one’s own opinions, and to critically think and answer questions proposed by others. Although the degree of participation varies among the students, students are more likely to be attracted and tied in learning for a longer attention span. Also, the pre-recorded video lecture before the flipped classroom provides more flexibility for students to decide when, where, and how many times they want to learn the lectures in order to best fit their personal learning style. These features of flipped classroom maximize the inclusiveness of the class. (4) Flipped classroom model is active and playful. Instead of passively and quietly listening to the instructor in the lectured-based classroom, students in the flipped classroom actively participate in the learning process, where presenting, listening, questioning, answering, discussing or even debating combine to make more fun for students. Therefore, students are more satisfied with the learning experience in the flipped classroom. In summary, our study showed that flipped classroom approach is generally very welcomed by the medical school students. This suggests that flipped classroom model may be a direction of education reform in medical student ophthalmology clerkship training.

Another interesting and enlightening finding from our study is that students evaluated the flipped glaucoma classroom less positively than they did the flipped ocular trauma classroom (Table 3). Consistently, teachers also agreed that ocular trauma is a more suitable topic for flipped classroom (Tables 4 and 5). Comparing to the ocular trauma flipped classroom, students in the flipped glaucoma classroom felt that the work load for preparation was too heavy, and that the class was overly theoretical and abstract. This can be caused by the differences between the clinical cases selected or the differences between instructors. However, one explanation is that glaucoma subject is more abstract in content and emphasizes more in memorization. Therefore, glaucoma subject can be more difficult for students to prepare by themselves for the presentation. For example, one challenge in learning glaucoma is the gonioscopy. Students reported that they need to spend much more time than they expected before the class. As a result, the challenge and pressure may overwhelm the interest and motivation from the flipped classroom. Similar results were reported by previous studies that, in the neuroanatomy course, which is also abstract and memorization-heavy, flipped classroom did not add value to the teaching process[11].On the contrary, the eye trauma subject is more concrete in contents, where clinical symptoms and signs are easier for students to understand. Students felt that flipped ocular trauma classroom was more beneficial. Our findings as well as others’ suggest that flipped classroom model may be better applied to those subjects that are less abstract in content and have a less emphasize in memorization. Also, the flipped classroom organizers and faculties should select suitable cases that intellectually challenge the students but not overwhelm them.

The flipped classroom model applied in this study was only a one-time teaching during the clerkship. Therefore, it is not surprising that the flipped classroom approach did not increase the scores of students in the final exam. Also, flipped classroom model may have certain limitations and its application needs to be optimized. First, the flipped classroom model may less clearly emphasize the key knowledge that students need to master. Therefore, the core knowledge needed for exam preparation may be diluted by the broad content in student-based discussion even with the control of instructors. Second, due to the “teaching” experience in the flipped classroom, students may feel overconfident for the exam and as a result prepare less for it. Third, the playful environment in the flipped classroom may lead students to a less serious attitude toward to the final exam. All these possible reasons may combine to make the fruit of flipped classroom less sweet, reflected by the comparable exam scores between the flipped classroom group and the lecture-based group. Our findings also indicated that the frequency of the flipped classroom, the cases selected and the way the discussion is led and controlled are all important factors need to be taken into consideration by educators in order to fully fulfill the advantages of the flipped classroom approach.

Another explanation for the comparable exam scores of students in flipped or non-flipped classroom may be due to the timing of test, which was performed right after either classroom model in this study. The flipped classroom approach may show greater advantages in helping students to form long-term memory due to students’ active learning[21, 22]. Therefore, it can be interesting, although beyond the scope of the current study, to reassess students’ performance on exams after a certain period of time without a lecture review before the test.

Last but not least, the performance in exam after flipped classroom did not increase may be also due to the intrinsic limitations of the evaluation system in the traditional exam. The way of delivery of knowledge is greatly evolved in the flipped classroom model; however, the way of evaluating the effectiveness of flipped classroom model does not evolve accordingly. The traditional exam format mainly uses multiple-choice questions to test students’ mastery of knowledge. However, the increase in students’ ability of critical thinking, self-learning, teamwork, presentation, and communication in the flipped classroom is overlooked by the current exam format. The Accreditation Council for Graduate Medical Education (ACGME) asks for six core competencies in medical training: patient care, medical knowledge, practice-based learning and improvement, interpersonal and communication skills, professionalism, and systems-based practice[23]. Compared to the traditional classroom, the flipped classroom model is a more efficient way to train students’ integrated competencies, which are essential for medical students in their transition into the upcoming real clinical work. Therefore, our findings also indicated that the current exam format may be out-of-date comparing to the newly-developed learning model, and that a reform in the evaluation system may be necessary to keep pace with the flipped classroom approach.

**Fig 3:**
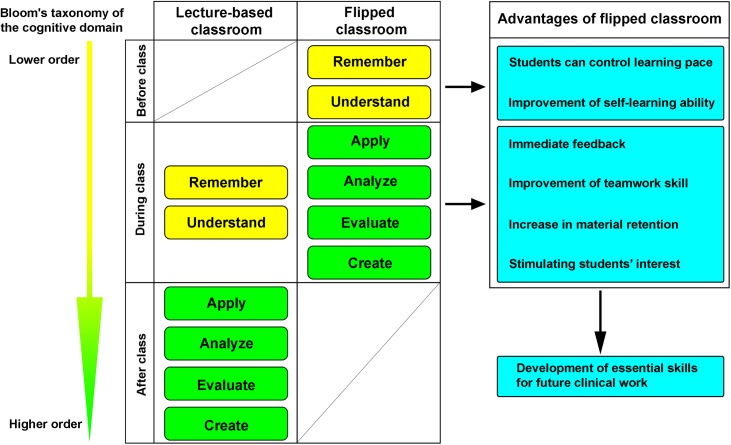
Bloom's taxonomy of the cognitive domains in traditional lecture-based classroom and flipped classroom. In the traditional non-flipped model, lower order levels of learning such as remembering and understanding are acquired in the class, and higher order levels of learning such as applying, analyzing, evaluating, and creating are achieved after the class. In the flipped classroom model, lower order levels of learning are acquired before the class, and higher order levels of learning are achieved during the class.

Taken together, the flipped classroom approach offers students opportunities to acquire lower order cognitive abilities such as remembering and understanding during before-class preparation, and use the valuable face-to-face in-class time for the development of higher order cognitive abilities including applying, analyzing, evaluating and creating as described by Bloom's taxonomy of the cognitive domains (Fig 3)[24, 25]. This transition not only provides more freedom and flexibility of self-paced learning, but also greatly improves team-working skill, increases material retention, and most importantly, stimulates students’ interest. The immediate feedback from the instructor also helps students to correct misconceptions. Further studies with larger student samples may be performed to optimize the subjects and frequency of flipped classroom in ophthalmology teaching. Also, a reform in the evaluation system may be required to better reflect the performance of medical students. We believe that with experience and optimization, the flipped classroom approach will be an effective and highly welcomed innovative learning method in diverse subjects in medical school. The stimulating, inclusive, active, playful, and student-centered learning environment in the flipped classroom will prepare medical students with a deeper understanding of the course material and help them better equipped with essential survival skills for future career development.
